# Cell-free hemoglobin increases inflammation, lung apoptosis, and microvascular permeability in murine polymicrobial sepsis

**DOI:** 10.1371/journal.pone.0228727

**Published:** 2020-02-03

**Authors:** Jamie E. Meegan, Ciara M. Shaver, Nathan D. Putz, Jordan J. Jesse, Stuart R. Landstreet, Han Noo Ri Lee, Tatiana N. Sidorova, J. Brennan McNeil, James L. Wynn, Joyce Cheung-Flynn, Padmini Komalavilas, Colleen M. Brophy, Lorraine B. Ware, Julie A. Bastarache

**Affiliations:** 1 Division of Allergy, Pulmonary and Critical Care Medicine, Vanderbilt University Medical Center, Nashville, TN, United States of America; 2 Departments of Pediatrics, Pathology, Immunology, and Experimental Medicine, University of Florida Health, Gainesville, FL, United States of America; 3 Division of Vascular Surgery, Vanderbilt University Medical Center, Nashville, TN, United States of America; 4 Department of Pathology, Microbiology and Immunology, Vanderbilt University Medical Center, Nashville, TN, United States of America; 5 Department of Cell and Developmental Biology, Vanderbilt University, Nashville, TN, United States of America; Ann and Robert H Lurie Children's Hospital of Chicago, Northwestern University, UNITED STATES

## Abstract

Increased endothelial permeability is central to the pathogenesis of sepsis and leads to organ dysfunction and death but the endogenous mechanisms that drive increased endothelial permeability are not completely understood. We previously reported that cell-free hemoglobin (CFH), elevated in 80% of patients with sepsis, increases lung microvascular permeability in an *ex vivo* human lung model and cultured endothelial cells. In this study, we augmented a murine model of polymicrobial sepsis with elevated circulating CFH to test the hypothesis that CFH increases microvascular endothelial permeability by inducing endothelial apoptosis. Mice were treated with an intraperitoneal injection of cecal slurry with or without a single intravenous injection of CFH. Severity of illness, mortality, systemic and lung inflammation, endothelial injury and dysfunction and lung apoptosis were measured at selected time points. We found that CFH added to CS increased sepsis mortality, plasma inflammatory cytokines as well as lung apoptosis, edema and inflammation without affecting large vessel reactivity or vascular injury marker concentrations. These results suggest that CFH is an endogenous mediator of increased endothelial permeability and apoptosis in sepsis and may be a promising therapeutic target.

## Introduction

Sepsis is a leading cause of morbidity and mortality in critically ill patients and is the most common cause of acute respiratory distress syndrome (ARDS) with an annual U.S. incidence of over 750,000, hospital costs of $24.3 billion, mortality of 25–30%, and high rates of long-term disability in survivors [1, 2]. There are currently no specific therapies for sepsis or sepsis-induced ARDS other than antimicrobials. Further, while it is well recognized that not all patients with severe infections develop sepsis and not all patients with sepsis develop ARDS, the underpinnings of this heterogeneity are not well understood. Our prior work has identified high levels of circulating cell-free hemoglobin (CFH) in 80% of patients with sepsis [3]. Moreover, sepsis patients with high circulating CFH levels have worse clinical outcomes and increased mortality compared to sepsis patients without elevations in CFH [3].

Hemoglobin circulates in vast quantities in the body but does so within the confines of the red blood cell which has a robust intracellular antioxidant system. When released from the red blood cell, hemoglobin is a potent pro-oxidant that can react with other proteins, lipids and DNA [4]. We have previously reported that CFH increases paracellular permeability in cultured endothelial cells [5, 6] and increases microvascular permeability in the *ex vivo* isolated perfused human lung [6] but the *in vivo* effects of CFH on the endothelium during sepsis have not been well studied. Increased microvascular permeability is a central feature of sepsis leading to depletion of intravascular volume as well as increased tissue edema, organ dysfunction and shock [7]. Given the role of CFH in increasing endothelial permeability [5, 6] and the elevated levels of CFH in the majority of sepsis patients [3], we hypothesized that release of CFH into the circulation during sepsis induces microvascular permeability leading to organ dysfunction and worse outcomes. To test this hypothesis, we augmented an established model of polymicrobial sepsis with CFH to reproduce the elevated levels of CFH that are observed in human sepsis. Further, we used *in vitro* models to determine the cellular mechanisms of CFH-mediated lung microvascular hyperpermeability.

## Methods

### Animals

All animal studies in this manuscript were reviewed and approved by the Vanderbilt University Medical Center Institutional Animal Care and Use Committee (Protocol Number: M1600006-01). In collaboration with institutional veterinarians we developed a care routine including frequent monitoring, nutritional supplementation, buprenorphine for pain alleviation, and humane endpoints to minimize suffering of mice in this study.

### Cecal slurry polymicrobial sepsis model

The cecal slurry peritonitis model has been described previously [8, 9]. Cecal slurry (CS) was prepared from 6-week-old female C57BL/6 mice purchased from The Jackson Laboratory (Bar Harbor, ME). Briefly, cecal contents were collected from euthanized donor mice, resuspended in 5% dextrose at 80 mg/mL, vortexed for 15 seconds, and filtered through a 25-gauge needle. Recipient 8-12-week-old male and female C57BL/6 mice were given intraperitoneal injection of CS at 1.7–2.0 mg/g body weight or 5% dextrose control. Purified LPS-free cell-free hemoglobin (CFH) was purchased from Cell Sciences (Cat. No. CSI9668A, Newburyport, MA), dissolved in PBS and sterile filtered prior to use. To test the independent effect of circulating CFH on organ dysfunction and outcomes during sepsis, recipient mice were also given retro-orbital injection of CFH (0.15 mg/g body weight) or PBS immediately after CS administration. This dose of CFH approximates the median concentration of CFH in human patients with sepsis [3, 10]. After injury induction, cage identifiers were covered to blind researchers to treatment groups for evaluation of sepsis severity. A series of clinical severity markers were used to determine the composite sickness score (protocol from Su et al [11] and Manley et al [12]): A) response to finger poke (4 = normal response, 3 = decreased response, 2 = severely decreased response, 1 = minimal response, 0 = no response (deceased)); B) signs of encephalopathy (4 = normal, 3 = tremors, staggering, 2 = twisting, 1 = turning and flipping, 0 = no response (deceased)); C) appearance (4 = normal, with one point subtracted for any of the following: piloerection, periorbital exudates, respiratory distress, diarrhea). During survival studies, mice were monitored every 4–6 hours for 96 hours and were immediately euthanized if immobility or a sepsis score equal to or less than 3 was observed. No animals required analgesia after sepsis induction. For other studies, mice were euthanized after 4 or 24 hours and samples were collected. Both CS and CS+CFH caused severe illness in mice that persisted through 24 hours. Mice that did not develop clinical signs of sepsis within 24 hours (those that never had sepsis score <12) were excluded from analysis in the CS and CS+CFH groups (5.6% of experimental mice).

### Sample collection

Mice were euthanized with pentobarbital. From a single, retro-orbital blood draw in heparinized tubes, whole blood was used for bacterial counts and plasma was prepared by centrifugation at 2,000 x g for 10 minutes. Bronchoalveolar lavage (BAL) was performed with 900 μL saline as previously described [13–15], centrifuged at 2,000 x g for 10 minutes, and supernatants were frozen. At the time of sacrifice, a peritoneal wash was performed with 5 mL of sterile PBS. The spleen was excised, dipped briefly in 100% ethanol to remove adherent bacteria, and homogenized in PBS. The aorta was removed and placed in heparinized Plasma-Lyte (10 units heparin/mL; Baxter Healthcare, Boston, MA) and immediately prepared for measurement of contractile function. Lungs were removed and immediately flash frozen in liquid nitrogen. All samples were stored at -80˚C until further analysis.

### Plasma cell-free hemoglobin

In one set of mice, blood was collected 4 hours post-injury and plasma CFH was measured using the HemoCue^®^ Plasma/Low Hb System (HemoCue America, Brea, CA) as previously reported [10, 16].

### Bacterial counts

Samples of whole blood, peritoneal wash, and homogenized spleen were serially diluted in PBS and plated onto LB agar, incubated at 37°C for 24 hours, and quantified. Data were natural log transformed prior to analysis. The limit of detection was 33 CFU/mL.

### Measurement of cytokines and endothelial markers

Interleukin 6 (IL-6), chemokine (C-X-C motif) ligand 1 *(*CXCL1) and tumor necrosis factor-α (TNF-α) were measured on the MSD electrochemiluminescence platform (Meso Scale Diagnostics, Gaithersburg, MD) [17]. Intercellular adhesion molecule 1 (ICAM-1), E-selectin, and plasminogen activator inhibitor-1 (PAI-1) were measured in duplicate using the Milliplex Magnetic Bead Panel Assay (Millipore Sigma, Burlington, MA).

### Measurement of aortic contractile function

Mouse aorta was cleaned and suspended in a muscle bath using 0.05 tungsten ring supports and adjusted to 0.3 g on a 2 g scale and equilibrated in a bicarbonate buffer (120 mM NaCl, 4.7 mM KCl, 1.0 mM MgSO_4_, 1.0 mM NaH_2_PO_4_, 10 mM glucose, 1.5 mM CaCl_2_, and 25 mM NaHCO_3_, pH 7.4), with 95% O_2_ / 5% CO_2_ at 37°C, and rinsed every 15 minutes maintaining a resting tension of 0.3 g for 1 hour. The aortic ring was manually stretched to 0.6 g followed by another 1 hour of equilibration without any washing as previously described [18–20]. After equilibration, the rings were contracted with 110 mM KCl with equimolar replacement of NaCl in bicarbonate buffer to determine smooth muscle functional viability. Contractile function was measured by treatment with phenylephrine (0.01–1 μM) to determine that 0.1 μM phenylephrine (PE) resulted in 60–70% KCl contraction, the dose chosen for future experiments. To determine endothelial-dependent relaxation, the tissue was then contracted with PE (0.1 μM) and relaxed with carbachol (CCH, 0.5 μM), an acetylcholine analogue [21]. Force measurements were obtained using the Radnoti force transducer (model 159901A, Radnoti LLC, Monrovia, CA) interfaced with a PowerLab data acquisition system and Chart software (AD Instruments Inc., Colorado Springs, CO) and were converted to stress by adjusting to the length and weight of the tissue. Percent relaxation was calculated as a change in stress compared to the maximal PE-induced contraction which was set as 100% as described previously [22, 23].

### Assessment of vascular permeability

In selected experiments, each mouse was given a retro-orbital injection of 100 μL AngioSense 750EX (2 nmol/100 μL; Perkin Elmer, Waltham, MA). Accumulation of AngioSense in excised lung tissue was measured using a Li-Cor Pearl camera. Plasma AngioSense fluorescence was measured using a fluorescence plate reader and did not differ between groups (p = 0.5786, data not shown). For lung wet weight to body weight ratio, the left lungs were excised and weighed. Lung wet weight/body weight was calculated according to published methods [24] by dividing the wet lung weight by the baseline body weight and normalizing for CS alone treatment.

### Lung histology and scoring

Lung sections (formalin-fixed and paraffin-embedded) were stained with hematoxylin and eosin as previously described [25, 26]. Ten nonoverlapping images per mouse were taken and scored by a blinded pulmonary pathologist by assessing septal thickening, edema, inflammation, and hemorrhage on a 1–5 scale [13].

### Lung apoptosis measurement

Terminal deoxynucleotidyl transferase dUTP nick end labeling (TUNEL) assay was performed with In Situ Cell Death Detection Kit, Fluorescein (Sigma Aldrich). Tissue sections were dewaxed and rehydrated according to standard protocol before incubating at 37°C for 30 minutes with 15 μg/mL Proteinase K. Slides were rinsed twice with PBS and incubated with TUNEL reaction mixture at 37°C in the dark for 1 hour. Slides were rinsed twice with PBS and mounted with DAPI mounting medium before confocal imaging with a Zeiss LSM880 laser scanning confocal microscope (Zeiss Germany). Endothelial cell apoptosis was ascertained using the Fluorescein In Situ Cell Death Detection Kit (Roche, Basel, Switzerland) in human lung microvascular endothelial cells (HULEC-5a, ATCC CRL-3244) and imaged on a Lionheart FX automated microscope (BioTek Instruments, Inc, Winooski, VT). TUNEL positive cells were counted by setting a GFP secondary mask within the DAPI primary mask to count the number of cells with co-localized staining.

### Lung cytokine mRNA expression

Whole lungs were excised, flash frozen and stored at -80˚C. mRNA was extracted from whole lungs using Qiagen RNeasy Plus Mini Kit (Hilden, Germany). cDNA was generated using a SuperScript VILO cDNA Synthesis Kit (Invitrogen, Carlsbad, CA). CXCL1 was quantified by qPCR and normalized to GAPDH expression using TaqMan primer probes (Thermo Fisher Scientific, Waltham, MA).

### Lung isoprostane measurements

F_2_ isoprostanes and isofurans were quantified in mouse lung homogenates by stable isotope dilution gas chromatography/negative ion chemical ionization mass spectrometry as described previously [27, 28].

### Statistical analysis

Statistical analysis was performed using GraphPad Prism (8.3.0) with α = 0.05. Statistical test performed for each figure is displayed in Table 1.

**Table 1:** Statistical analysis.

Figure	N =	Statistical Test	Graph
1A	9–14	One-way ANOVA + Tukey’s mc	Mean + 95% CI
1B	20–24	Kruskal-Wallis + Dunn’s mc	Median + 95% CI
1C	3–7	Log-rank (Mantel-Cox)	Survival proportions
1D-E	7–10	Kruskal-Wallis + Dunn’s mc	Median + 95% CI
2A-C	11–12	Mann-Whitney	Median + 95% CI
3A-C	10–22	Kruskal-Wallis + Dunn’s mc	Median + 95% CI
3D	5	Kruskal-Wallis + Dunn’s mc	Median + 95% CI
4B	9–10	Mann-Whitney	Median + 95% CI
4C	19–21	Mann-Whitney	Median + 95% CI
4E	6–7	Bonferroni-Dunn	Median + 95% CI
5B	8–10	One-way ANOVA + Tukey’s mc	Mean + 95% CI
5C	12	Mann-Whitney	Median + 95% CI
6A	11–12	Mann-Whitney	Median + 95% CI
6B	16	Unpaired t test	Mean + 95% CI
7A-B	6	Mann-Whitney	Median + 95% CI

Abbreviations: mc = multiple comparison; CI = confidence interval

## Results

### CFH increases severity of illness and mortality in sepsis

**Fig 1:**
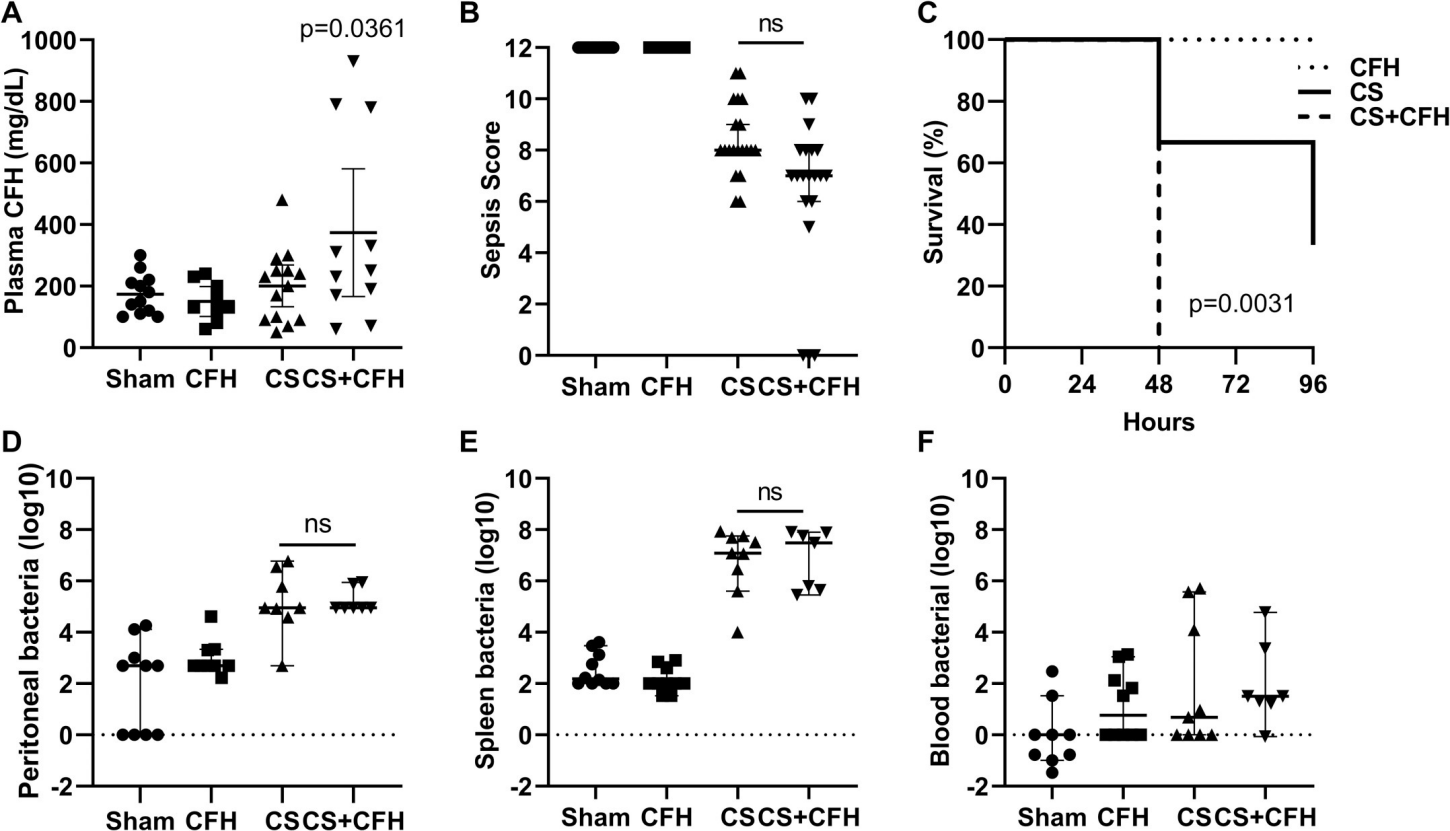
Intravenous CFH augments mortality in sepsis. To establish a clinically relevant model of sepsis with elevated circulating cell-free hemoglobin (CFH), we treated mice with an intravenous (IV) injection of CFH, an intraperitoneal (IP) injection of CS, simultaneous IV CFH and IP CS, or sham IV and IP injections. (A) After 24 hours, the CS+CFH group had the highest circulating CFH levels (n = 9–14, p = 0.0361 vs. Sham). The CS+CFH group had a (B) slightly lower sepsis score indicating more severe illness (n = 20–24, p = ns vs. CS) and (C) lower survival (n = 3–7, p = 0.0031). Bacterial counts measured in (D) peritoneal wash, (E) spleen homogenates and (F) blood were elevated in both CS and CS+CFH groups but did not differ between these two groups (n = 7–10).

Injection of a single dose of IV CFH immediately following CS injection resulted in increased circulating CFH in plasma at 24 hours (Fig 1A) establishing that circulating CFH is elevated in our model as it is in 80% of human patients with sepsis. Although our CFH dosing was calculated to achieve circulating CFH levels similar to those measured in patients with sepsis (10–40 mg/dL) [3, 10], the actual levels achieved were higher. CS+CFH had a trend towards increased severity of illness (Fig 1B) and a significant increase in mortality (Fig 1C) compared to CS alone suggesting that CFH contributes to severity of illness and mortality in this sepsis model. The increased severity of illness and mortality was not due to increased bacterial burden in the peritoneal space (Fig 1D) or to changes in bacterial dissemination into the circulation (Fig 1E and 1F) since the bacterial counts at these sites did not differ between CS and CS+CFH groups.

### CFH increases circulating inflammatory markers

**Fig 2:**
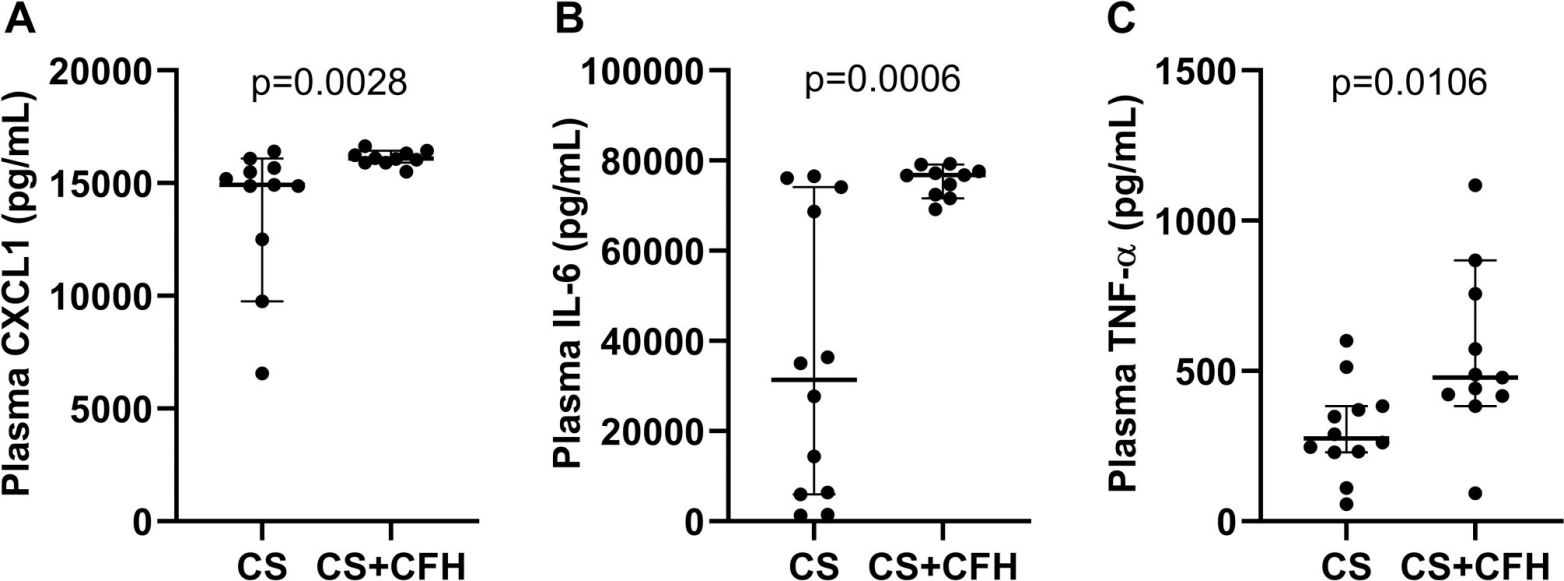
Intravenous CFH augments systemic cytokine increases in murine sepsis. Plasma cytokines were measured 24 hours after CS or CS+CFH treatment (n = 11–12). (A) CXCL1 (p = 0.0028), (B) IL-6 (p = 0.0006) and (C) TNF-α (p = 0.0106) were higher in CS+CFH versus CS alone.

Systemic inflammation is a hallmark of sepsis. To test the contribution of circulating CFH to systemic inflammation, we measured plasma IL-6, CXCL1 and TNF-α in mouse plasma at 24 hours after treatment with CS or CS+CFH. We found that all three cytokines and chemokines were increased in CS+CFH versus CS alone (Fig 2) suggesting that CFH augments systemic inflammation in sepsis.

### CFH does not affect circulating markers of endothelial injury or large vessel endothelial function

**Fig 3:**
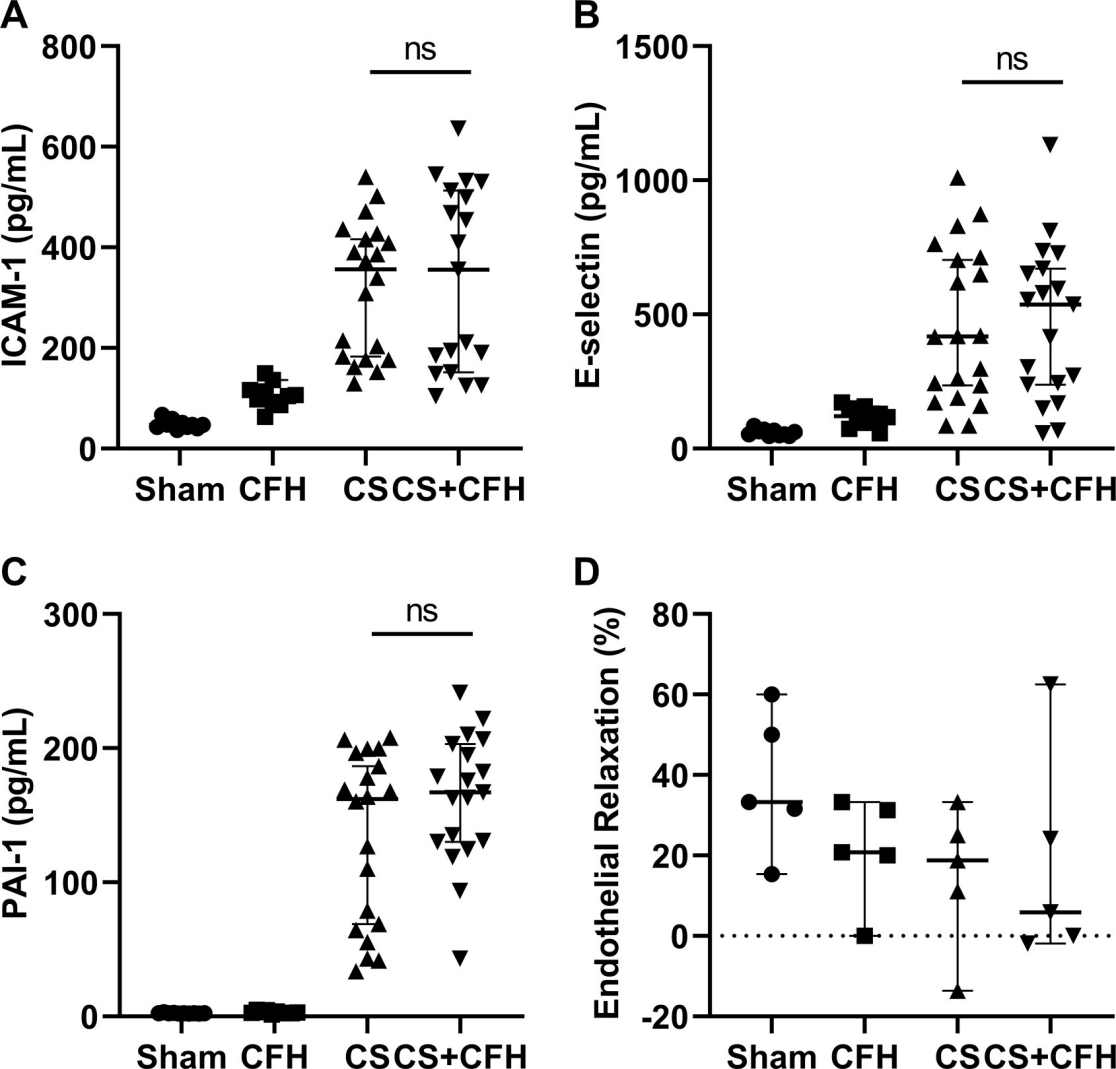
CFH causes vascular injury in murine sepsis. Markers of endothelial injury (n = 10–22), (A) ICAM-1, (B) E-selectin, and (C) PAI-1 were measured 24 hours after CS or CS+CFH treatment. All three markers are elevated in CS and CS+CFH groups but do not statistically differ from each other. (D) To test whether large vessel injury was induced by CFH, we measured aortic relaxation in response to acetylcholine in excised aortas 24 hours after treatment. CFH did not worsen aortic relaxation when combined with CS treatment (n = 5).

Endothelial dysfunction is central to the pathogenesis of sepsis and is characterized by both macro- and microvascular dysfunction. Markers of endothelial injury are elevated in patients with sepsis and correlate with severity of illness and organ dysfunction [29–31]. To determine whether CFH increases endothelial injury in sepsis, we measured ICAM-1, E-selectin, and PAI-1 at 24 hours in mouse plasma (Fig 3A–3C). All three markers increased in CS and CS+CFH but did not statistically differ between the two. We aimed to test the impact of CFH on both macro- (aortic relaxation) and microvascular (lung permeability) endothelial dysfunction. To assess whether this was a result of large vessel endothelial injury, we measured aortic relaxation in freshly excised mouse aortae 24 hours after CS or CS+CFH treatment. Although aortic endothelial-dependent relaxation is impaired after induction of sepsis with CS, CFH did not cause additional impairment of aortic relaxation when combined with CS suggesting that CFH does not affect large vessel endothelial function during sepsis (Fig 3D).

### CFH increases lung microvascular permeability

**Fig 4:**
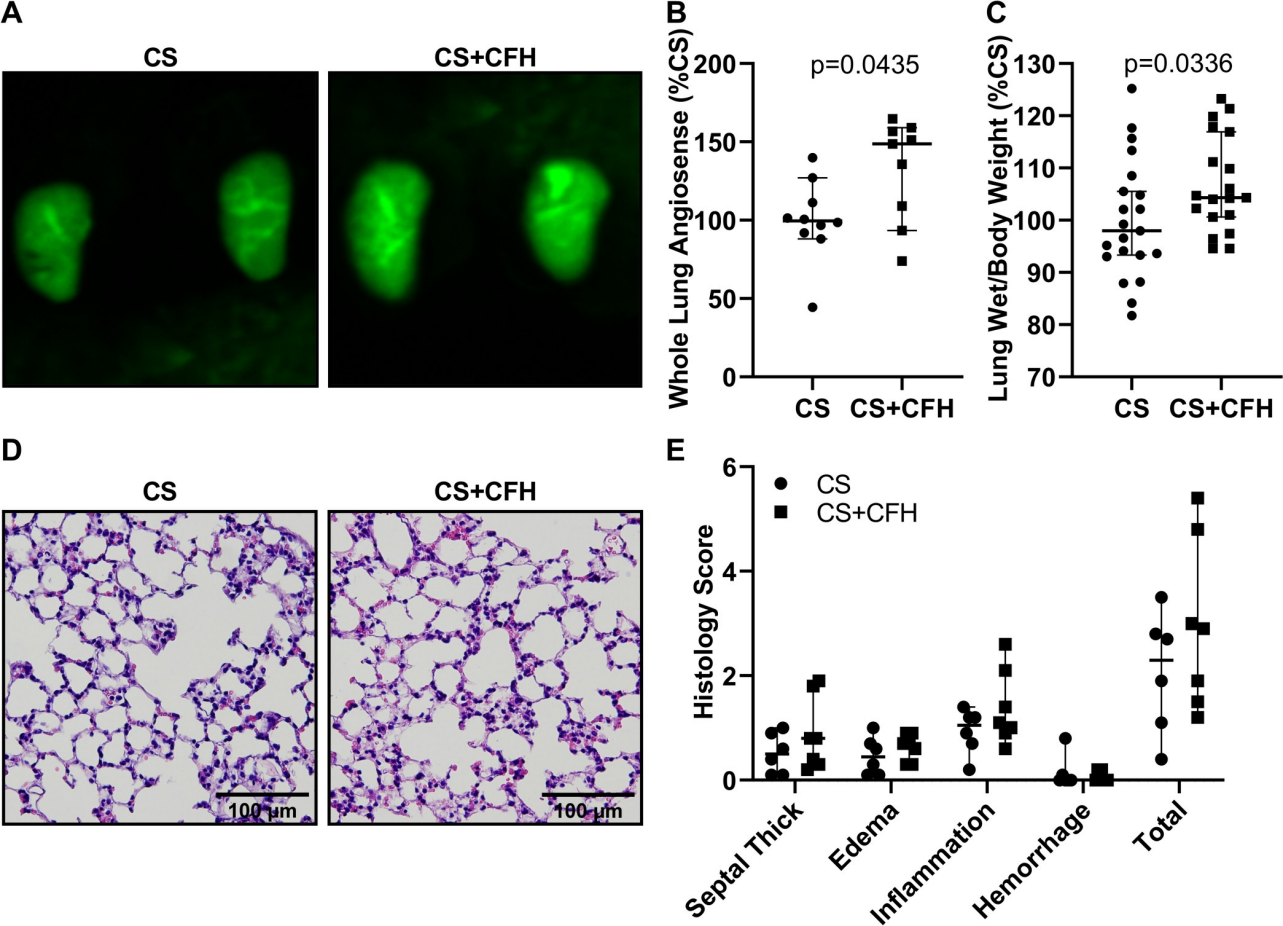
CFH increases lung edema formation in murine sepsis. Lung microvascular permeability was measured by accumulation of fluorescent AngioSense in mouse lungs 24 hours after injection of sepsis. (A) Excised lungs were imaged using a high sensitivity camera. (B) Fluorescence intensity was quantified and was higher in CS+CFH versus CS alone (n = 9–10, p = 0.0435). (C) To measure lung fluid accumulation, we calculated lung wet weight to mouse body weight, expressed as a percent of CS treatment, and found that CS+CFH increases lung weight versus CS alone (n = 19–21, p = 0.0336). (D-E) Lung histology showed slightly increased septal thickening, edema, and inflammation in CS+CFH versus CS alone but was not statistically different (n = 6–7).

To next test how CFH may contribute to microvascular endothelial injury, we used a fluorescently tagged 70 kD macromolecule, AngioSense, to assess microvascular permeability in the lung. We found that whole lung AngioSense accumulation was significantly higher at 24 hours in CS+CFH compared to CS alone (Fig 4A and 4B). In addition, lung wet weight to body weight was higher in CS+CFH versus CS alone, suggesting increased pulmonary edema in the presence of CFH (Fig 4C). BAL albumin levels were not different between CS and CS+CFH (not shown). Notably, lung histology showed no differences in septal thickening, edema, inflammation, or hemorrhage between groups (Fig 4D and 4E).

### CFH increases lung apoptosis

**Fig 5:**
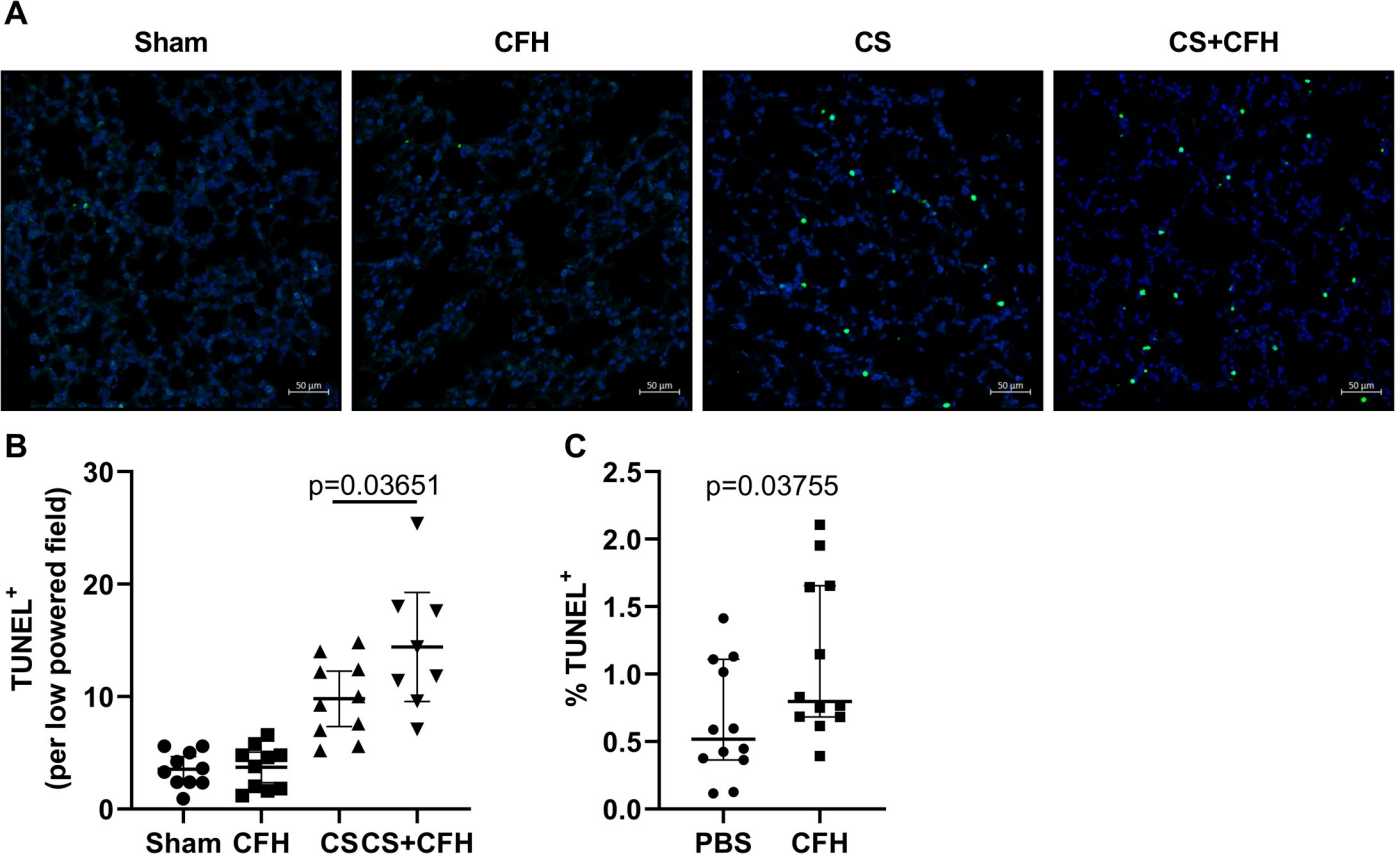
CFH increases lung endothelial apoptosis in sepsis. (A) Formalin fixed mouse lung tissue was stained for TUNEL (green) with nuclear DAPI staining (blue). (B) The number of TUNEL positive cells was higher in CS+CFH versus CS alone by blinded quantification of low powered images (n = 8–10, p = 0.03651). (C) We also assessed apoptosis by TUNEL staining of HULECs in culture and found that CFH (1 mg/mL) treatment induced apoptosis at 4 hours (n = 12, p = 0.03755).

Endothelial apoptosis contributes to increased microvascular permeability in sepsis [32]. To test whether CFH-mediated endothelial apoptosis is one mechanism by which CFH increases lung inflammation and permeability during sepsis, we measured lung apoptosis in mice treated with CS or CS+CFH at 4 hours. We stained lung sections for TUNEL to indicate apoptotic cells (Fig 5A). At 4 hours, the number of apoptotic cells in the lung was significantly higher in CS+CFH compared to CS alone. We counted TUNEL positive cells (Fig 5B) by blinded assessment. To test whether CFH could specifically induce apoptosis of lung endothelial cells, we measured apoptosis by TUNEL staining of cultured primary human lung endothelial cells (HULEC) treated with CFH or PBS for 4 hours (Fig 5C). In culture, CFH induces lung microvascular endothelial apoptosis.

### CFH increases lung inflammation

**Fig 6:**
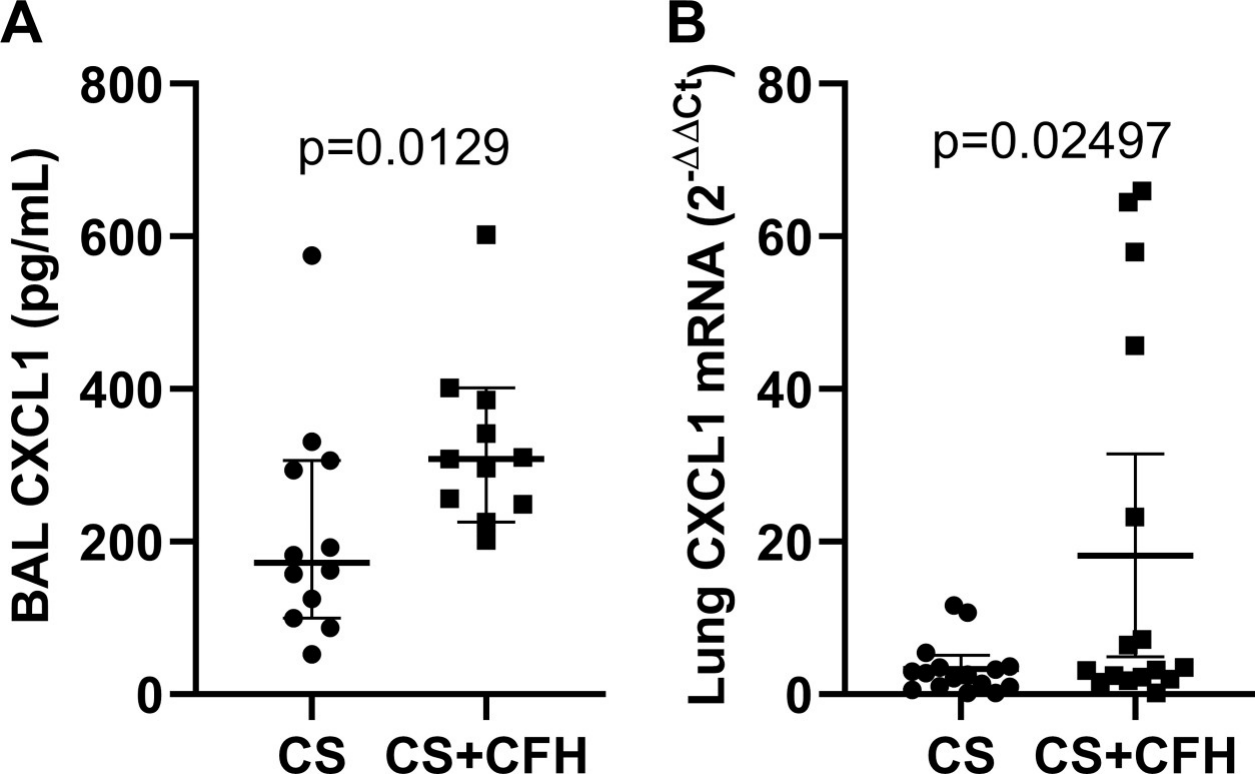
CFH increases lung inflammatory cytokine expression. (A) CXCL1 measured by ELISA increased in mouse BAL collected 24 hours after induction of sepsis in CS+CFH versus CS alone (n = 11–12, p = 0.0129). (B) Lung CXCL1 expression measured by PCR in whole lungs at 24 hours also increased in CS+CFH versus CS alone (n = 16, p = 0.02497).

To test whether CFH also augments lung inflammation, we measured BAL CXCL1 (Fig 6A) as well as whole lung mRNA expression of CXCL1 (Fig 6B). In sepsis, CFH augments both CXCL1 mRNA and protein expression in the lung.

### CFH increases lung oxidative injury

**Fig 7:**
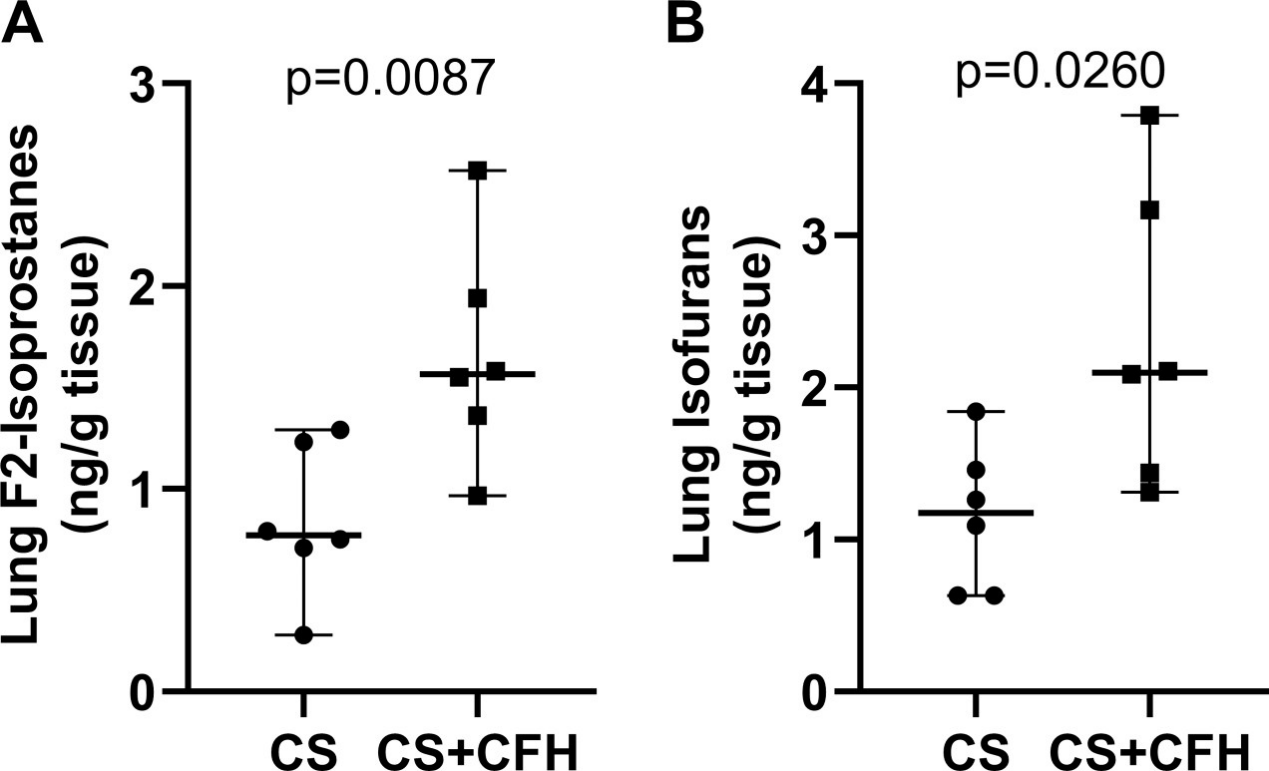
CFH increases lung oxidative injury. (A) Whole lung F_2_-isoprostanes (n = 6, p = 0.0087) and (B) isofurans (n = 6, p = 0.0260) increased at 24 hours in CS+CFH versus CS alone.

CFH is known to cause oxidative injury in a variety of cells and tissues. To test whether CFH increased oxidative injury in the lungs in this sepsis model, we measured F_2_-isoprostanes and isofurans in flash frozen lungs at 24 hours. We found that CS+CFH increases both F_2_-isoprostanes (Fig 7A) and isofurans (Fig 7B) compared to CS alone suggesting that CFH increases oxidative injury in the lung.

## Discussion

In this manuscript we describe a new model of polymicrobial sepsis in which we can test the independent effects of CFH in mediating sepsis pathogenesis. Importantly, we have designed the model such that CS alone does not increase circulating CFH and increasing CFH requires a single dose of IV CFH. The detriments of CFH seem to require an inflammatory environment, as CFH injection alone does not have an effect. The major finding of these studies is that increased circulating CFH to model the increased CFH seen in 80% of sepsis patients, independently increased endothelial injury, vascular permeability, systemic inflammation, organ injury, severity of illness and death in a clinically relevant murine sepsis model. Addition of a single dose of IV CFH to the CS polymicrobial sepsis model was associated with an increase in lung TUNEL positive cells but we were not able to determine whether these cells were endothelial in origin. Overall, these results show that CFH augments several indices of systemic and lung injury during sepsis.

Recent work highlights the central role of the endothelium in sepsis [7]. We found that endothelial injury markers such as ICAM-1, E-selectin, and PAI-1 were elevated to a similar degree in CS+CFH versus CS alone, suggesting that plasma levels of these markers may not reflect microvascular changes at the tissue level. In studying large vessel endothelial function, we found that while sepsis alone did have a trend towards decreased vasorelaxation compared to sham or CFH, there was no difference between CS and CS+CFH suggesting that CFH does not affect macro-vascular endothelial function. Interestingly, the deleterious effects of CFH in our study seem to be limited to the microvascular endothelium. These data were somewhat surprising in light of other studies showing inhibition of aortic relaxation by hemoglobin [33–35]. It is important to note that prior studies were done in isolated aortic tissue exposed to CFH *ex vivo*, a key difference from our study. When mice were treated with CFH alone there was no effect on vascular reactivity, perhaps because intact, healthy animals can rapidly clear CFH as the low CFH levels in this group would imply. In fact, the persistence of CFH at 24 hours in the CS+CFH group suggests that in the setting of sepsis there is persistence of circulating CFH. While not the focus of this work, it will be important to measure circulating CFH over time to test this hypothesis in future studies.

In contrast, we did observe increased microvascular permeability in CS+CFH versus CS alone. Increased microvascular permeability is a defining feature of sepsis that leads to organ injury and dysfunction. In patients, microvascular injury in the lung manifests as acute respiratory distress syndrome (ARDS) characterized by increased microvascular permeability, lung inflammation, and oxidative injury. To test whether CFH augments lung injury during sepsis, we measured lung microvascular permeability by tissue accumulation of AngioSense, a 70 kD fluorescently labeled macromolecule. Interestingly, while AngioSense accumulation was greater in CS+CFH treated mice, BAL albumin was not different suggesting that AngioSense accumulated in the lung interstitium and not the airspace. This finding suggests that while there was a breakdown of the lung microvascular endothelial barrier, the alveolar epithelial barrier remained intact. The increased lung wet weight-to-body weight ratio with CS+CFH in the absence of increased BAL albumin supports this concept. Foundational studies in the 1970s showed that the epithelial barrier is relatively resistant to albumin flux, accounting for >92% of total alveolar-capillary barrier resistance [36] suggesting that an endothelial directed insult, such as CFH in our model, could induce endothelial but not epithelial permeability. Recent studies have identified endothelial apoptosis as a critical mechanism of lung endothelial permeability in sepsis [32] but the drivers of endothelial apoptosis in sepsis are still unclear. Here we show that increased lung apoptosis is an early event in sepsis, occurring at 4 hours after CS+CFH treatment. In addition, CFH increased sepsis-induced lung apoptosis suggesting an independent role for CFH. The induction of lung apoptosis may explain the increase in lung AngioSense accumulation in the absence of elevated BAL albumin.

In this study, a single dose of IV CFH significantly increased sepsis severity of illness and mortality. One possible explanation for this could be that CFH facilitates growth and dissemination of intra-peritoneal bacteria. However, we were unable to detect any significant differences in bacterial burden in the peritoneal wash, spleen, or blood of CS and CS+CFH animals. It remains possible that there are differences in numbers of anaerobic organisms not detected by our culture methods. Furthermore, we found no evidence that CFH facilitated bacterial growth by providing an iron source.

Another possible mechanism by which CFH augments disease severity would be through increased inflammation. Despite the lack of effect on bacterial burden, CFH increased systemic pro-inflammatory cytokines but the specific mechanism of increased cytokine release in response to CS+CFH is not clear. In addition, CFH augments lung inflammation as measured by increased CXCL1 in the BAL fluid and lung tissue. Lung inflammation is a key feature of clinical ARDS. Interestingly, we did not see an increase in BAL neutrophils which is somewhat surprising given the elevated CXCL1, a major chemoattractant for neutrophils. Again, this may be explained by a relative preservation of the alveolar epithelial barrier which could limit inflammatory cell influx into the airspace. Alternatively, CFH may affect the timing of inflammatory cell influx, a possibility that needs to be further explored in the future. Studies have shown a delay between peak BAL CXCL1 (24 hours) and peak BAL neutrophil count (48–72 hours) in response to intratracheal LPS [37]. In addition, others have shown a discordance between CXCL1 and BAL cell counts with a high CXCL1 and low BAL cell count in murine models of sepsis or endotoxemia [38, 39] suggesting that lung CXCL1 expression may not correlate with BAL cell counts in indirect models of lung injury.

Finally, we found that CFH independently increases lung F_2_-isoprostanes and isofurans, markers of increased oxidant stress, in this polymicrobial sepsis model. Increased oxidant stress in the lung during sepsis has been well described in cecal ligation and puncture models [40–43] but none of these studies examined CFH as a potential mediator of oxidant injury. The CLP model of sepsis also has elevated circulating CFH, raising the possibility that some of its oxidant effects could be due to CFH [44]. Our novel polymicrobial sepsis model with controlled supplementation of CFH allowed us to specifically test the mechanistic contribution of CFH during sepsis. CFH is highly susceptible to oxidation, resulting in oxidized ferric (Fe^3+^) or ferryl (Fe^4+^) CFH [45]. In a study of post-operative coronary artery bypass patients, pericardial fluid contained high levels of oxidized CFH that correlated with high levels of F_2_-isoprostanes [46]. In addition, we have shown that treatment of human microvascular endothelial cells with CFH depletes intracellular ascorbate, a major cellular antioxidant [5], and a study by Goldman et al showed that oxidized CFH induces apoptosis and cytotoxicity in bovine aortic endothelial cells [47]. Furthermore, we have previously shown that targeting ferryl CFH with acetaminophen in sepsis patients results in decreased F_2_-isoprostanes, suggesting that ferryl CFH is driving the oxidant injury [10]. Since oxidative stress is a major mediator of inflammation, it is possible that CFH-induced increases in oxidative stress adds to the pro-inflammatory and pro-apoptotic burden that lead to tissue injury and represent an integral cause of microvascular endothelial dysfunction during sepsis.

In summary, we have shown that induction of circulating levels of CFH that mimic levels observed in human sepsis has independent effects on endothelial permeability, systemic and lung inflammation and pulmonary microvascular apoptosis in the setting of polymicrobial intra-abdominal sepsis. These effects of CFH involve an increased inflammatory response and increased oxidant stress, which may underlie mechanisms leading to increased endothelial apoptosis. These findings add to the growing body of literature on cellular and molecular mechanisms of microvascular hyperpermeability and organ dysfunction in sepsis. Further, as increased CFH is found in 80% of sepsis patients, therapies that target CFH represent a novel approach to personalized sepsis care.
